# Effect of Bacteria from the Genus *Azospirillum* on Oxidative Stress Levels in Wheat *Triticum aestivum* L. in the Presence of Copper, Nickel, and Lead

**DOI:** 10.3390/microorganisms13020334

**Published:** 2025-02-04

**Authors:** Maria V. Gureeva, Marina S. Kirillova, Veronika A. Trandina, Vera A. Kryukova, Anna A. Eremina, Alina A. Alimova, Margarita Y. Grabovich, Artem P. Gureev

**Affiliations:** 1 Department of Biochemistry and Cell Physiology, Voronezh State University, 394018 Voronezh, Russia; marine-2002_13@mail.ru (M.S.K.); veronikasafrosenko@gmail.com (V.A.T.); margarita_grabov@mail.ru (M.Y.G.); 2 Department of Genetics, Cytology and Bioengineering, Voronezh State University, 394018 Voronezh, Russia; vera.kryukova2017@yandex.ru (V.A.K.); anya.kryyy@gmail.com (A.A.E.); aa10022607@gmail.com (A.A.A.); gureev@bio.vsu.ru (A.P.G.)

**Keywords:** *Azospirillum*, wheat, heavy metals, plant growth-promoting rhizobacteria, copper, nickel, lead

## Abstract

Heavy metals (HMs) exert a negative impact on physiological processes in plants, which can adversely affect the productivity of agricultural crops. In this experiment, we assessed the potential to mitigate the toxic effects of HMs on soft wheat through the use of rhizospheric microorganisms from the genus *Azospirillum*. In the initial phase of the experiment, we identified the most resistant *Azospirillum* strains to Cu (from 1.5 to 15 mg/L), Ni (from 2 to 20 mg/L), and Pb (from 15.9 to 159.4 mg/L). Both Ni and Pb significantly inhibited bacterial growth and induced substantial oxidative stress in the majority of the studied strains. The strain *A. picis* B-2897^T^ exhibited the highest resistance to all HMs. The cultivation of wheat in soil supplemented with Cu led to an increased growth rate and enhanced wheat productivity. Conversely, Ni and Pb reduced wheat productivity by 65% and 27%, respectively. This was accompanied by chlorophyll depletion and a decrease in the expression of genes *NDOR* and *GST*, which are involved in xenobiotic detoxification. Pre-inoculation of seeds with *Azospirillum* led to a decrease in HM concentration in the plant seedlings; in particular, *A. picis* B-2897^T^ reduced the level of Ni from 0.005% to a concentration below the detectable level (i.e., below 0.001%), and Pb from 0.014% to 0.008%. The bacteria stimulated the expression of genes responsible for xenobiotic detoxification and contributed to the increased growth and productivity of wheat. Thus, *Azospirillum* can be utilized as a bioproduct to alleviate the toxic effects of HMs.

## 1. Introduction

Wheat (*Triticum* spp.) is a vital cereal grain with a global production of over 770 million tonnes [1]. Wheat plays a critical role in global food security, being cultivated on over 217 million hectares annually [2]. Wheat provides nearly 55% of carbohydrates and daily protein for 85% of the world’s population [3], yet abiotic stresses pose a serious threat to wheat production worldwide [4].

Among various abiotic stresses, heavy metal (HM) accumulation is one of the major agronomic problems that seriously threaten food safety [5]. In many developed and developing countries, HM pollution has increased due to rapid industrialization, the long-term use of untreated wastewater for irrigation, and intensive agricultural practices [5]. High levels of HMs in soil can damage plant root systems and disrupt the photosynthesis process and membrane permeability, thereby limiting plant growth and reducing crop yield and quality [6].

HM exposure in plants can lead to the generation of reactive oxygen species (ROS), such as H_2_O_2_, hydroxyl radical (OH^−^), and superoxide anion radical (O_2_^−^), disrupting the cellular redox homeostasis. This redox imbalance can result in HM toxicity in plants [7]. Additionally, HMs can bind to oxygen, nitrogen, and sulfur atoms inside cells; thus, HMs inactivate enzymes by binding to cysteine residues [8].

It should also be noted that the toxicity of different HMs to plants varies. This is due to the fact that some HMs, such as nickel (Ni) and copper (Cu), are trace elements essential for plant growth and are part of the active centers of enzymes. Accordingly, their low concentrations even stimulate plant growth. In contrast, other HMs, such as lead (Pb), are not essential for plant growth and are toxic at lower concentrations [7]. In addition, high concentrations of Pb in the soil environment minimize the availability of other essential nutrients for plants (Ca, Fe, Mg, Mn, P, and Zn) by blocking the penetration of ions or their binding to the ion carrier [8].

Recently, some HM-tolerant plant growth-promoting rhizobacteria (PGPRs) have been found to improve plant growth in metal-contaminated soils and reduce the bioavailability and uptake of hazardous metals by plants, resulting in safe food production [6]. Metal-tolerant microbes, when used as microbial inoculants (biofertilizers), have been reported to detoxify metal toxicity by reducing the bioavailability of metals in soil and converting them to less toxic forms [5]. Microorganisms can employ different mechanisms to counteract HM toxicity. These include exopolysaccharide secretion, biotransformation, metal oxidation, electrostatic interaction, methylation and demethylation, and complexation [9]. Moreover, some HM-resistant PGPRs can reduce HM toxicity in plants by altering the physiological status of plants. For example, some PGPRs can enhance host plant tolerance to HM stress by stimulating the antioxidant defense system of plants [10]. Bacteria of the genus *Azospirillum* are among the best-characterized PGPRs [11]. In particular, studies have been conducted on the mitigation of oxidative stress caused by copper and lead in wheat by these bacteria [12,13]. *Azospirillum brasilense* EMCC1454 can tolerate chromium stress up to 260 μM and exhibit various growth-promoting activities in chickpea (*Cicer arietinum* L.) plants exposed to different levels of chromium stress [14]. *Azospirillum lipoferum* (UAP154 and UAP40) has been used as a remediation agent for copper toxicity in alfalfa (*Medicago sativa* L.) seeds [15]. Thus, studies on the effect of *Azospirillum* on wheat resistance to HMs have been conducted only for two species, but currently, the genus *Azospirillum* includes 25 species with validly published names (https://lpsn.dsmz.de/genus/azospirillum), and the metabolic potential of this genus to protect plants against HMs has not been fully studied. In a number of studies, it has been found that representatives of the genus *Azospirillum* are resistant to HMs; however, identification down to the species level was not carried out [16,17,18]. To expand our understanding of the potential of individual representatives of the genus *Azospirillum* to protect plants against HMs, we evaluate the protective effect of six strains of different *Azospirillum* species and one strain of *Niveispirillum* (until 2014, this strain also belonged to the genus *Azospirillum*) and select the most promising among them in terms of reducing the toxic effect of copper, nickel, and lead on wheat.

The aim of this work was to select *Azospirillum* strains most resistant to Cu, Ni, and Pb and to evaluate their effect as bioinoculants on the metabolism and yield of wheat in the presence of the corresponding HMs. In the first stage of the work, the most resistant *Azospirillum* strains to each HM were selected, and changes in the level of oxidative stress markers were studied in these strains during incubation on media with the addition of HMs. In the second stage of the study, these strains were inoculated onto wheat seeds before germination in soil containing HMs. At this stage, the potential of the inoculants to improve the growth characteristics of wheat and reduce oxidative stress and dysfunctions of the photosynthetic apparatus, as well as their effect on the bioavailability of HMs and the expression of genes responsible for phases I and II of xenobiotic detoxification, were assessed. In the third stage, a field experiment was conducted to assess the effect of *Azospirillum* inoculants on wheat productivity indicators.

## 2. Materials and Methods

### 2.1. Cultivation of Bacteria

The following bacterial strains were used in this work: *Azospirillum thiophilum* B-2513^T^, *A. brasilense* B-1547^T^, *A. picis* B-2897^T^, *Niveispirillum irakense* (basonym *A. irakense*) B-2893^T^, *A. halopraeferens* DSM 3675^T^, *A. lipoferum* B-1519^T^, and *A. baldaniorum* B-3036^T^ (=Sp245^T^). *A. thiophilum* B-2513^T^ was cultivated in our laboratory. *A. brasilense* B-1547^T^, *A. picis* B-2897^T^, *N. irakense* B-2893^T^, *A. lipoferum* B-1519^T^, and *A. baldaniorum* B-3036^T^ (Sp245^T^) were obtained from the All-Russian Collection of Microorganisms (VKM), and *A. halopraeferens* DSM 3675^T^ was obtained from the Leibniz Institute DSMZ—German Collection of Microorganisms and Cell Cultures GmbH (DSMZ). All *Azospirillum* strains were grown in liquid modified peptone-succinate-salt (MPSS) medium containing the following components (g/L): (NH_4_)_2_SO_4_, 1.0; CaCl_2_ 2H_2_O, 0.03; MgSO_4_ 7H_2_O, 1.0; sodium succinate, 1.0; peptone, 2.0 and solutions of vitamins and microelements [19]; pH 7.5. Bacteria were cultivated in flasks with cotton plugs for 3 days in a liquid nutrient medium of the composition indicated above at a temperature of 29 °C. To test the resistance of bacterial strains to HMs, HM salts in the required concentrations were added to the flasks with the nutrient medium before autoclaving (Table 1). Bacterial growth was assessed by measuring the optical density value determined on a PE-5400UF spectrophotometer (Ecros, Saint Petersburg, Russia) at a wavelength of 600 nm. The purity of bacterial preparations was monitored using the microscope Olympus CX41 (Olympus, Tokio, Japan).

For biochemical studies, the cells were pelleted by centrifugation for 10 min at 11,000 g in a Centrifuge 5804R (Eppendorf, Hamburg, Germany) and washed three times with 0.1 M Tris-HCl buffer, pH 7.5. Then, the cells resuspended in the buffer were disrupted in an ultrasonic disintegrator UZDN-2T (Academpribor, Moscow, Russia) in an ice bath for 1.5 min at a power of 500 W and a frequency of 22 kHz. To obtain the supernatant, the disrupted cells were pelleted for 10 min at 11,000 g at 4 °C. The supernatant was used to determine catalase activity and MDA (malondialdehyde) concentration.

To treat wheat seeds, the cell suspension was prepared as described above and diluted with saline to a concentration of 10^8^ cells/mL.

### 2.2. Plant Cultivation

The soft spring wheat *Triticum aestivum* L., strain Kurier RS1, was used as a model organism. Wheat seeds were obtained from the National Grain Center named after P.P. Lukyanenko, Krasnodar, Russia. During the greenhouse experiment, the plants were grown on 10 cm diameter Petri dishes with autoclaved soil weighing 12.5 g. Plants were grown under continuous lighting conditions (16/8 h light/dark cycle) at a constant temperature of 24 °C and a relative humidity of at least 40%. HM salts in the required concentrations were introduced into the Petri dishes by watering. Treatment with a bacterial suspension was carried out for 20 min before sowing, after which the seeds were dried and sown on Petri dishes.

For biochemical studies, the roots and shoots of 7-day-old wheat sprouts were homogenized using a Bio-prep-6 Homogenizer (Allsheng, Hangzhou, China) in phosphate-buffered saline (Rosmedbio, Saint Petersburg, Russia). For DNA and RNA extraction, homogenization was performed using the same device in lysis buffers from the corresponding kits.

### 2.3. Field Experiment

**Figure 1:**
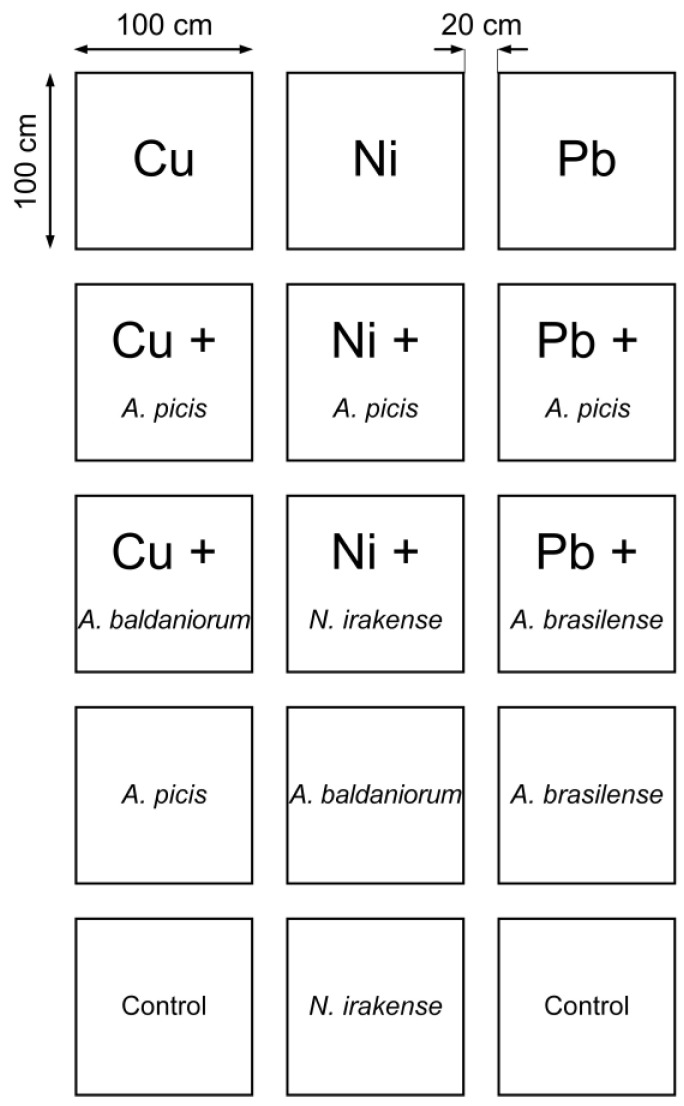
Planting scheme of experimental wheat groups in a field experiment. Chemical element symbols on the scheme indicate that the corresponding HM was introduced into this plot in a concentration equal to the MAC. The name of the bacterial species on the scheme indicates that the seeds were inoculated in suspensions of the corresponding bacteria before planting in the soil in this plot.

The field experiment was conducted from 27 April to 25 July 2024 on chernozem soil in Voronezh, Russia (51°35′21.0” N, 39°09′22.7” E) (Figure 1). Plants were grown in small plots of 1 m^2^, with a distance of at least 20 cm between plots. HMs were added to the topsoil at a depth of 20 cm in a concentration corresponding to the maximum allowable concentration (MAC) in the form of salts, and the soil was mixed with HMs. In mature wheat, the mass of all seeds collected from a plot, the mass of 1000 seeds, the number of ears per plot, the number of grains per ear, the length of the ears, the height of the shoots, and the total mass of straw and roots were determined.

### 2.4. Assay of Catalase Activity

Catalase activity was determined at a wavelength of λ = 230 nm by measuring the decrease in hydrogen peroxide concentration, following the method described by [20], using a PE-5400UF spectrophotometer (Ecros, Saint Petersburg, Russia). Protein content was determined using the Lowry method [21].

### 2.5. Assay of MDA Concentration

The concentration of MDA was determined using a spectrophotometric method based on the reaction of MDA with thiobarbituric acid at 100 °C in an acidic medium, followed by measurement of the optical density of the reaction mixture at 532 nm [22], using a PE-5400UF spectrophotometer (Ecros, Saint Petersburg, Russia).

### 2.6. Assay of Pigments Level

The levels of chlorophylls and β-carotene in the shoots of 7-day-old wheat sprouts were estimated spectrophotometrically using a Hitachi U-2900 spectrophotometer (Hitachi, Tokio, Japan). To determine the level of chlorophylls, 100 mg of sprouts were homogenized in 2 mL of acetone and then centrifuged for 5 min at 5000 g. The optical density of the supernatant was measured at wavelengths of 645 nm and 663 nm. To determine the level of β-carotene, an extract was prepared from mechanically homogenized plant material in acetone, and then the optical density was measured at 470 nm [23].

### 2.7. Determination of Gene Expression Levels

Total RNA was isolated using the commercial ExtractRNA kit (Evrogen, Moscow, Russia) according to the provided protocol. cDNA was synthesized using the commercial REVERTA-L kit (AmpliSens, Moscow, Russia) using the isolated RNA template. The samples were incubated in a thermocycler for 30 min at 37 °C, after which PCR was performed with the obtained cDNA using qPCRMix (Evrogen, Moscow, Russia) and 1X SYBR Green Master Mix (BioDye, Saint Petersburg, Russia). PCR conditions were as follows: 3 min at 95 °C, then 95 °C for 10 s, 59 °C for 30 s, and 72 °C for 30 s, which were repeated for 40 cycles. Primers for the amplification reaction were selected using the Primer-BLAST program. All primers are presented in Table 2. The *gapdh* gene was used as a reference.

### 2.8. Assay of Heavy Metal Content

Dried wheat biomass was ground in liquid nitrogen. The content of HMs in plants was determined using an S8 Tiger X-ray fluorescence spectrometer (Bruker AXS GmbH, Karlsruhe, Germany). The results were processed using the developed methods in the Spectra Plus program, the Organics calibration method (Bruker AXS GmbH, Karlsruhe, Germany). The accuracy of the analysis was controlled using TR-1 (GSO 8922-2007).

### 2.9. Statistical Analysis

Statistical analysis was performed using the STATISTICA 12 software package (StatSoft, Inc., Tulsa, OK, USA). The results are presented as means ± S.E.M. The normality of distribution was assessed using the Shapiro–Wilk test. Since the distribution deviated from normal, non-parametric statistical methods were used to evaluate the significance of differences between groups. The statistical significance of differences between the two groups was assessed via the Mann–Whitney U test. When analyzing three or more groups, the Kruskal–Wallis test was used. Statistical significance was considered to be *p* < 0.05.

## 3. Results

### 3.1. Resistance of Bacterial Strains to Heavy Metals

**Figure 2:**
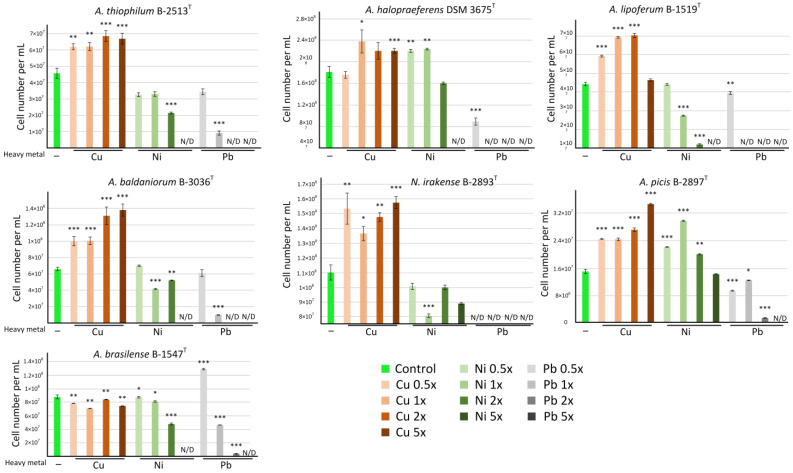
Resistance of the studied bacteria to Cu, Ni, and Pb. Control, no HMs were added to nutrient medium; 0.5×, the corresponding metal was added to nutrient medium in concentration corresponding to 0.5 MAC (see Table 1); 1×, the corresponding metal was added to nutrient medium in concentration corresponding to 1 MAC (see Table 1); 2×, the corresponding metal was added to nutrient medium in concentration corresponding to 2 MAC (see Table 1); 5×, the corresponding metal was added to nutrient medium in concentration corresponding to 5 MAC (see Table 1). All measurements were conducted with at least 10 technical replicates. Values are given as mean ± SEM. * *p* < 0.05, ** *p* < 0.01, and *** *p* < 0.001 compared to control (M-U test).

To assess the toxicity of HMs in relation to bacterial strains, we used metal concentrations corresponding to 0.5, 1, 2, and 5 MAC per kg of soil and added the same amounts per liter of nutrient medium (Table 1). All the studied bacteria demonstrated the highest resistance to Cu. Cu (from 1.5 to 15 mg/L) even had a stimulating effect on all the studied strains except for *A. brasilense* B-1547^T^ (Figure 2).

Ni was the second most toxic metal. Only two of the studied strains, *N. irakense* B-2893^T^ and *A. picis* B-2897^T^, withstood a Ni concentration corresponding to 5 MAC (20 mg/L). Moreover, in the case of *A. picis* B-2897^T^, Ni concentrations from 0.5 MAC (2 mg/L) to 2 MAC (8 mg/L) stimulated bacterial growth (*p* < 0.01), and at 5 MAC (20 mg/L), the growth was at the control level. Ni also had a stimulating effect on *A. halopraeferens* DSM 3675^T^ at concentrations of 0.5 MAC (2 mg/L) and 1 MAC (4 mg/L) (*p* < 0.01). For the remaining strains, Ni concentrations from 0.5 MAC (2 mg/L) to 2 MAC (8 mg/L) either did not have a significant effect on bacterial growth or reduced it (Figure 2).

Pb had the greatest inhibitory effect on bacterial growth. None of the strains could withstand a concentration of 5 MAC (159.4 mg/L). Only two strains, *A. picis* B-2897^T^ and *A. brasilense* B-1547^T^, showed very weak growth at a Pb concentration of 2 MAC (63.8 mg/L). For *A. thiophilum* B-2513^T^ and *A. baldaniorum* B-3036^T^, the maximum tolerated Pb concentration was 1 MAC (31.9 mg/L); for *A. halopraeferens* DSM 3675^T^ and *A. lipoferum* B-1519^T,^ it was 0.5 MAC (15.9 mg/L), and the growth of *N. irakense* B-2893^T^ was completely inhibited at any Pb concentration used in the study (Figure 2).

For further studies, for each HM, the two most resistant strains were selected. *A. picis* B-2897^T^ and *A. baldaniorum* B-3036^T^ were the most resistant for Cu, *A. picis* B-2897^T^ and *N. irakense* B-2893^T^ were the most resistant for Ni, and *A. picis* B-2897^T^ and *A. brasilense* B-1547^T^ were the most resistant for Pb.

### 3.2. MDA Concentration and Catalase Activity in Different Azospirillum Strains

Catalase activity and MDA concentration were determined in the most resistant strains at maximum tolerated concentrations of HMs. For Cu, these were *A. picis* B-2897^T^ and *A. baldaniorum* B-3036^T^, which withstood concentrations of 5 MAC (15 mg/L); for Ni, these were *A. picis* B-2897^T^ and *N. irakense* B-2893^T^, which withstood concentrations of 5 MAC (20 mg/L), and for Pb, these were *A. picis* B-2897^T^ and *A. brasilense* B-1547^T^, which withstood concentrations of 1 MAC (31.9 mg/L).

Pb increased the MDA content in *A. picis* B-2897^T^ by approximately 1.7 times (not statistically significant). For copper, it increased by eight times, and for nickel, by thirteen times (both *p* < 0.05). Pb increased the MDA content in *A. brasilense* B-1547^T^ by approximately two times (*p* < 0.05). Ni increased the MDA content in *N. irakense* B-2893^T^ by two times (*p* < 0.01). However, Cu did not have an impact on the MDA content in *A. baldaniorum* B-3036^T^ (Table 3).

**Figure 3:**
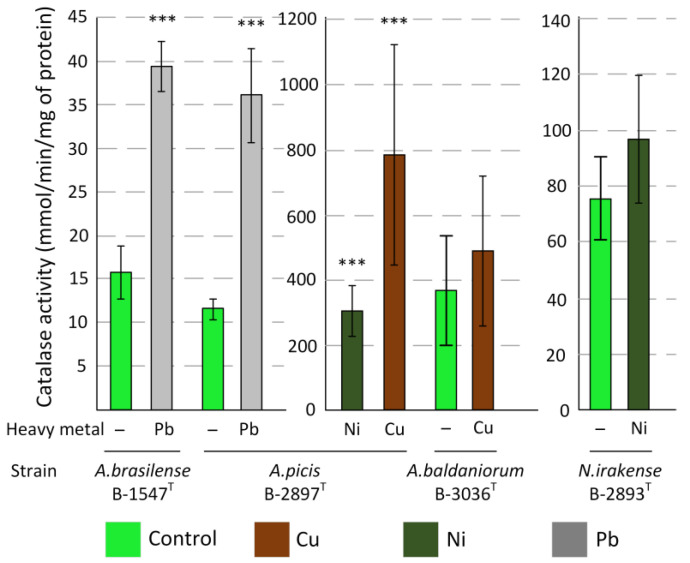
Effect of HMs on catalase activity in *Azospirillum* strains. Values are given as mean ± SEM. All measurements were conducted with at least three technical replicates. *** *p* < 0.001 compared to control (M-U test).

An addition of HMs did not result in statistically significant changes in catalase activity in *A. baldaniorum* B-3036^T^ and *N. irakense* B-2893^T^. Pb increased catalase activity nearly three-fold (*p* < 0.001) in *A. brasilense* B-1547^T^. All HMs significantly increased catalase activity in *A. picis* B-2897^T^: Pb three-fold, Ni twenty-six-fold, and Cu sixty-eight-fold (all *p* < 0.001) (Figure 3).

### 3.3. Wheat Growth and Oxidative Stress in a Petri Dish Experiment

To assess the effect of HMs on the growth characteristics of wheat, 7-day-old seedlings grown on sterile soil in Petri dishes in the presence of a salt of the corresponding metal at a concentration corresponding to the MAC in the soil were used. To evaluate the effect of bacteria, wheat seeds were incubated in a bacterial suspension with a concentration of 10^8^ cells/mL for 20 min before sowing. For each HM, two of the most resistant strains were used. The height and weight of shoots, as well as the weight of roots, were measured.

HMs did not have a statistically significant effect on the height of wheat shoots, while preincubation of wheat seeds with *N. irakense* B-2893^T^ and *A. brasilense* B-1547^T^ increased shoot height by 17% and 16%, respectively (both *p* < 0.05). Treatment of wheat seeds grown in the presence of Ni salts with *A. picis* B-2897^T^ resulted in an increase in shoot height by 19% relative to the control (*p* < 0.01) (Table S1).

**Figure 4:**
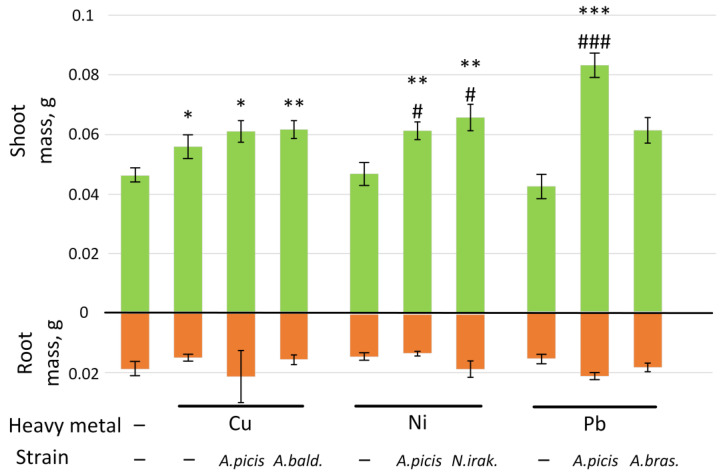
Effect of HMs and bacterial inoculation on the mass of roots and shoots of 7-day-old wheat seedlings. *A.picis*, the seeds were treated with *A. picis* B-2897^T^ before sowing; *A. bald*., the seeds were treated with *A.baldaniorum* B-3036^T^ before sowing; *N.irak*., the seeds were treated with *N. irakense* B-2893^T^ before sowing; *A.bras*., the seeds were treated with *A. brasilense* B-1547^T^ before sowing; Cu, Ni, Pb, and salts of the corresponding metals were added to the soil at a concentration equal to the MAC. Values are given as the average over 10 measurements ± SEM. * *p* < 0.05, ** *p* < 0.01, and *** *p* < 0.001 compared to control; # *p* < 0.05 and ### < 0.001 in the sample with metals and bacteria relative to the sample with metals.

The presence of Cu in the soil increased the mass of shoots by 21% (*p* < 0.05), while preincubation with *A. picis* B-2897^T^ and *A. baldaniorum* B-3036^T^ increased it by 32% (*p* < 0.05) and 33% (*p* < 0.01), respectively. Ni did not affect shoot weight. Preincubation of seeds with *A. picis* B-2897^T^ and *N. irakense* B-2893^T^ grown in soil supplemented with Ni increased shoot weight by 33% and 42%, respectively (both *p* < 0.01 compared to control, *p* < 0.05 compared to Ni only). The addition of Pb to soil did not significantly decrease shoot weight, whereas preincubation of seeds with *A. picis* B-2897^T^ significantly increased shoot weight by 80% (*p* < 0.001 compared to control and Pb only). Preincubation of seeds with *A. brasilense* B-1547^T^ did not change the weight of aboveground plant parts. Growing wheat in the presence of HM salts and preincubating seeds with plant growth-promoting bacteria did not result in statistically significant changes in root mass (Figure 4). No statistically significant changes in MDA content in wheat roots and shoots were observed under the influence of HMs or plant growth-promoting bacteria (Table S2).

### 3.4. Effect of Heavy Metal and Bacterial Inoculation on the Pigment Content

**Figure 5:**
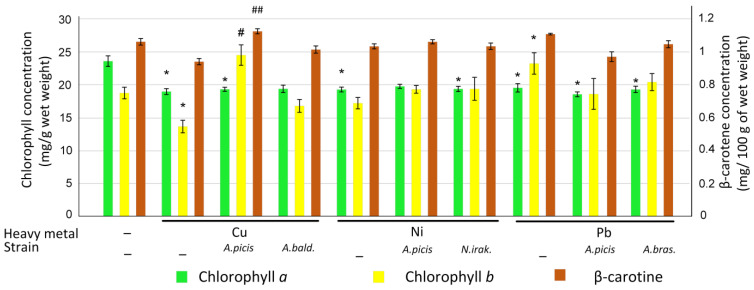
Effect of HMs in the soil and seeds pre-treatment with a suspension of bacterial cells on the content of chlorophyll *a*, chlorophyll *b*, and β-carotene in 7-day-old wheat seedlings. *A.picis*, the seeds were treated with *A. picis* B-2897^T^ before sowing; *A. bald*., the seeds were treated with *A. baldaniorum* B-3036^T^ before sowing; *N.irak*., the seeds were treated with *N. irakense* B-2893^T^ before sowing; *A.bras*., the seeds were treated with *A. brasilense* B-1547^T^ before sowing; Cu, Ni, Pb, and salts of the corresponding metals were added to the soil at a concentration equal to the MAC. Values are given as mean ± SEM. The number of biological replicates for each group was at least 10. All measurements were conducted with at least 3 technical replicates.* *p* < 0.05 compared to control, # *p* < 0.05, and ## < 0.01 in the sample with metals and bacteria relative to the sample with metals.

The content of total chlorophyll, chlorophyll *a*, chlorophyll *b*, and β-carotene in the shoots of 7-day-old wheat seedlings was determined. All three HMs reduced the content of chlorophyll *a* (all *p* < 0.05). Only Cu reduced the concentration of chlorophyll *b* (*p* < 0.05), while Pb, on the contrary, stimulated an increase in the concentration of chlorophyll *b* relative to the control (*p* < 0.05). At the same time, in those wheat seeds that were pre-inoculated with *A. picis* B-2897^T^ and *A. brasilense* B-1547^T^ and grown on soil with the addition of Pb, an increase in the concentration of chlorophyll *b* relative to chlorophyll *a* was not detected. At the same time, inoculation of wheat seeds with *A. picis* B-2897^T^ prevented the Cu-induced decrease in the concentration of chlorophyll *b* in wheat shoots (Figure 5).

HMs did not cause changes in β-carotene levels relative to the control. However, treatment of *A. picis* B-2897^T^ in the presence of Cu increased β-carotene levels relative to wheat grown in the presence of Cu without bacterial inoculation (*p* < 0.01) (Figure 5).

### 3.5. Effect of Heavy Metal and Bacterial Inoculation on the Gene Expression

**Figure 6:**
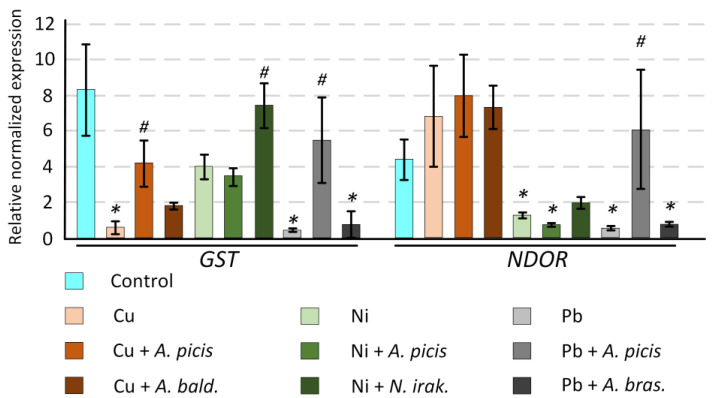
Effect of HMs in the soil and pre-treatment of seeds with bacterial cell suspension on the normalized level of *GST* and *NDOR* gene transcripts in 7-day-old wheat seedlings. *A.picis*, seeds were treated with *A. picis* B-2897^T^ before sowing; *A. bald*., seeds were treated with *A. baldaniorum* B-3036^T^ before sowing; *N.irak*., seeds were treated with *N. irakense* B-2893^T^ before sowing; *A.bras*., seeds were treated with *A. brasilense* B-1547^T^ before sowing; Cu, Ni, Pb, and salts of the corresponding metals were added to the soil at a concentration equal to the MAC. Values are given as mean ± SEM. The number of biological replicates for each group was at least 10. All measurements were conducted with at least 3 technical replicates. * *p* < 0.05 compared to control, # *p* < 0.05, and comparison of the sample with metals and bacteria relative to the sample with metals.

In our study, we examined the expression of the genes encoding glutathione-S transferase (GST) and NADPH-dependent diflavin oxidoreductase (NDOR) in the roots of 7-day-old seedlings. These enzymes participate in the detoxification of xenobiotics. Cu caused a significant decrease in *GST* expression (~15-fold, *p* < 0.05) but did not affect *NDOR* expression. Preincubation of wheat seeds with *A. picis* B-2897^T^ partially prevented the Cu-dependent decrease in *GST* gene expression (Figure 6).

Ni, on the contrary, contributed to the decrease in *NDOR* gene expression but did not affect *GST* expression. However, in the group of wheat seedlings treated with *N. irakense* B-2893^T^, *GST* expression was twice as high as in wheat seedlings grown only in soil supplemented with Ni (*p* < 0.05). Additionally, *N. irakense* B-2893^T^ prevented a Ni-induced decrease in *NDOR* expression, with the differences from the control being statistically insignificant (Figure 6).

Pb addition to soil significantly decreased the expression of both genes (both *p* < 0.05). *A. picis* B-2897^T^ significantly increased it: 1.5 times above the control level for *NDOR* and almost to the control level for *GST* (both *p* < 0.05) (Figure 6).

### 3.6. Impact of Bacteria Inoculation on the Heavy Metal Content in the Biomass of Wheat

**Figure 7:**
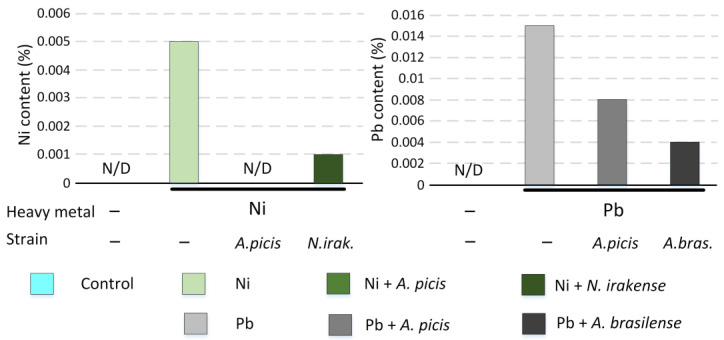
Content of HMs in dry mass of wheat grown on soil with added HMs, as well as with inoculation of seeds with bacteria. *A.picis*, seeds were treated with *A. picis* B-2897^T^ before sowing; *N.irak*., seeds were treated with *N. irakense* B-2893^T^ before sowing; *A.bras*., seeds were treated with *A. brasilense* B-1547^T^ before sowing; Ni, Pb, and salts of the corresponding metals were added to the soil in a concentration equal to the MAC. Values are given as % of the metal content from the total dry mass of wheat sprouts grown on Petri dishes.

The addition of 1 MAC Ni (4 mg/kg) to soil resulted in nickel content in the dry biomass of wheat being 0.005%, while in control samples, Ni was not detected. However, in sprouts of wheat grown in soil with Ni addition but with seeds previously inoculated with *A. picis* B-2897^T^, the Ni level was below the detectable limit (i.e., below 0.001%). Inoculation of seeds with *N. irakense* B-2893^T^ also prevented Ni accumulation, and its content was 0.001% (Figure 7).

The introduction of 1 MAC (31.9) of Pb into the soil caused an increase in its level in dry biomass to 0.014%. In the control samples, Pb was not detected. Inoculation of seeds with *A. picis* B-2897^T^ resulted in wheat accumulating only 0.008% Pb, and when inoculating seeds with *A. brasilense* B-1547^T^, the Pb level in the dry mass of wheat was 0.004% (Figure 7).

The sensitivity of the device used did not allow Cu detection in the samples.

### 3.7. Effect of Heavy Metal and Bacterial Inoculation on the Wheat’s Harvest

**Figure 8:**
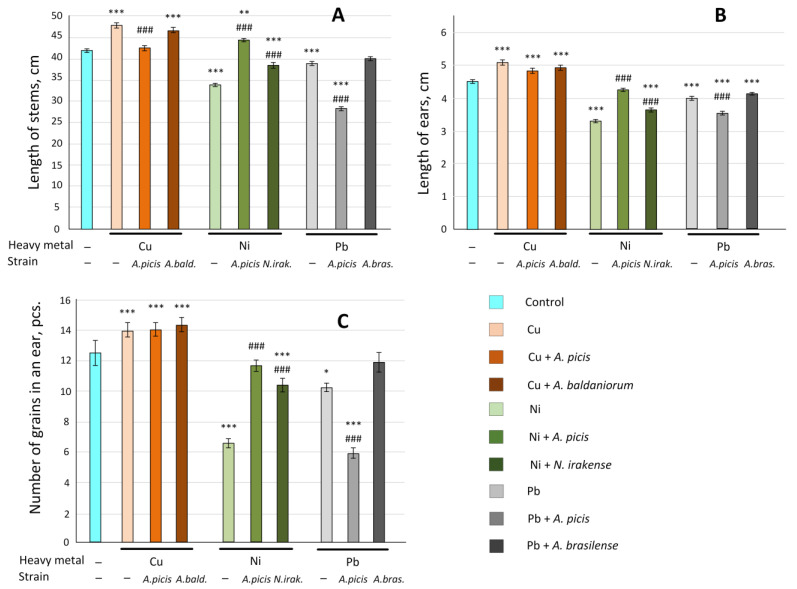
Effect of HMs and bacterial inoculation on wheat yield parameters in a field experiment. (**A**): length of stems; (**B**): length of ears; (**C**): number of grains in an ear. C, control; *A.picis*, seeds were treated with *A. picis* B-2897^T^ before sowing; *A. bald*., seeds were treated with *A. baldaniorum* B-3036^T^ before sowing; *N.irak*., seeds were treated with *N. irakense* B-2893^T^ bacteria before sowing; *A.bras*., seeds were treated with *A. brasilense* B-1547^T^ bacteria before sowing; Cu, Ni, Pb, and salts of the corresponding metals were added to the soil at a concentration equal to the MAC. Values are given as the average for all wheat sprouts ± SEM. At least 500 seeds were planted in each plot. * *p* < 0.05, ** *p* < 0.01, and *** *p* < 0.001 relative to control, and ### *p* < 0.001 in the sample with metals and bacteria relative to the sample with metals.

Cu improved growth and yield parameters in wheat. The average stem length and ear length increased by 13%, and the number of grains per ear increased by 10% (all *p* < 0.05) (Figure 8). The total seed weight and 1000 seed weight increased by 15%. The total straw weight increased by 41%, and the root system weight more than doubled. Bacterial inoculation, in most cases, did not affect growth and yield parameters (Table S3).

Ni addition to the soil resulted in a 20% decrease in the average stem length, a 27% decrease in the ear length, and a nearly two-fold decrease in the number of grains per ear (all *p* < 0.001) (Figure 8). Ni also contributed to a decrease in the total seed weight from 142 to 50 g per 1 m^2^. At the same time, seed inoculation with *A. picis* B-2897^T^ contributed to an increase in the total yield by more than three times. Inoculation with *N. irakense* B-2893^T^ increased by 2.3 times compared to the plot in which only Ni was added (Table S3). Ear length was increased by 28% and 10%, respectively (both *p* < 0.001), and the number of grains per ear was increased by 78% and 58%, respectively (both *p* < 0.001) (Figure 8).

Pb reduced the total seed weight by 27%, although the weight of 1000 seeds was, on the contrary, 6% higher than the control. Pb significantly suppressed the development of the root system (by 66%) (Table S3) and reduced the number of grains in an ear by 19% (*p* < 0.001) (Figure 8). Inoculation of wheat seeds with *A.picis* B-2897^T^ when grown on soil with the addition of Pb, on the contrary, even aggravated the toxic effect of this HM. *A. brasilense* B-1547^T^ did not have a statistically significant effect on the growth and yield of wheat compared to the group grown on soil with the addition of Pb only (Figure 8, Table S3).

## 4. Discussion

*Azospirillum* has varying degrees of resistance to various HMs. Their resistance to various HMs can be ranked in the following order: Cu > Ni > Pb. In general, our data are consistent with previously obtained results. It has been shown that the soil microbial community was tolerant to Cu, Ni, and Zn but not tolerant to Pb [24]. It was shown that for *A. brasilense* Sp245 (at present, this strain is classified within the species *A. baldaniorum* [25]) 0.2 mM concentrations of NiSO_4_·7H_2_O (56.17 mg/L), CuSO_4_·5H_2_O (50 mg/L), and Pb(NO_3_)_2_ (66.24 mg/L) do not inhibit the bacterial growth substantially [26]. The data we obtained for this strain are consistent with those reported in [26].

For *A. baldaniorum* Sp245^T^, it was shown that the minimum inhibitory concentration of Cu^2+^ is 0.9 mmol/L (57.6 mg Cu/L or 225 mg CuSO_4_·6H_2_O/L) [27], which does not contradict our results. In our experiment, the maximum tested concentration of Cu^2+^ was 0.23 mmol/L (5 MAC per kg of soil), and at this concentration, bacterial growth was not suppressed but rather activated (Figure 2). Most of the *Azospirillum* strains used in our study tolerated Ni concentrations of 2 MAC (8 mg Ni/L) but were inhibited at 5 MAC (20 mg Ni/L) (Figure 2). Representatives of the genus *Azospirillum* were previously shown to be tolerant to Ni in this concentration range: they were present at the cathode and anode in a single-chamber microbial electrolytic cell with a Ni concentration of 12.5 mg/L used for Ni removal from water [28].

One of the factors that can inhibit bacterial growth is oxidative stress caused by HMs. One of the most commonly used markers of oxidative stress is the MDA assessment [29]. Our results show that for most of the bacterial strains we studied, the HM concentrations used caused oxidative stress. The only exception was *A. baldaniorum* B-3036^T^, in which, on the contrary, the amount of MDA decreased in the presence of Cu (Table 3). The activity of antioxidant enzymes can also be a marker of oxidative stress. Catalase dismutates H_2_O_2_ into oxygen and water, which plays an important role in protecting cells from oxidative damage by H_2_O_2_ [30]. Pb exerts the maximum toxic effect on bacterial cells and significantly increases catalase activity in all strains studied. At the same time, *A. baldaniorum* B-3036^T^ and *N. irakense* B-2893^T^ do not experience oxidative stress in the presence of Cu and Ni since no changes in catalase activity were noted. This is consistent with the data on MDA concentration in *A. baldaniorum* B-3036^T^.

At a concentration of 1 MAC (3 mg/kg soil), Cu did not have a negative effect on the growth characteristics of wheat; on the contrary, it increased the length of the sprouts on the seventh day after planting (Figure 4). In addition, Cu enhanced the agricultural productivity of wheat in a field experiment (Figure 8). It has been previously shown that soil application of Cu in greenhouse experiments promotes an increase in growth characteristics and productivity of wheat (maximum effect at 1.5 mg/kg soil). The authors attribute this positive effect to the ability of Cu to reduce Zn and Fe concentrations in plants [31]. However, soil application of Cu contributed to a decrease in chlorophyll concentration in wheat sprouts (Figure 5), which was previously noted in wheat plants growing in a Cu-contaminated area [32]. In addition, Cu reduced the expression of the *GST* gene in wheat roots (Figure 6), which mediates the conjugation of various xenobiotics, including HMs, with GSH [33]. It was previously shown that GST activity was reduced in the presence of cadmium and arsenic in *Phragmites* and *Typha latifolia* plants. It was suggested that this may be due to the use of HMs as alternative substrates [34]. Inoculation of wheat seeds with *A. picis* B-2897^T^ during wheat cultivation on Cu-supplemented soil increased the expression of the *GST* gene (Figure 6). It has been previously shown that the introduction of various hormones, in particular jasmonic acid, can promote an increase in the level of *GST* transcripts in the roots, which contributes to protection against Cu-induced stress [35]. It is known that various PGPRs, including *Azospirillum*, can stimulate the production of hormones by plants [36]. In addition, *A. picis* B-2897^T^ stimulated an increase in the concentration of chlorophyll *b.* The content of β-carotene was increased in the shoots of this group of plants (Figure 5), which also indicates a positive effect of this strain of microorganisms on the photosynthetic apparatus of 7-day-old wheat seedlings. *A. baldaniorum* B-3036^T^, which, according to microbiological studies, is also a Cu-resistant strain (Figure 2), did not induce such positive changes.

Ni is an essential micronutrient (at low concentrations of 0.01–5 μg/g dry weight) for plants. Ni(II) is a functional component in urease, glyoxalases, peptide deformylases, methyl Co-M reductases, hydrogenases, and superoxide dismutases. However, high Ni concentrations (>10^−4^ M/L) can cause toxicity symptoms and growth inhibition in most plants. Ni at 870 mg/kg significantly inhibited the germination of wheat seedlings compared to the uninoculated control [37]. In our study, Ni (4 mg/kg soil) did not affect the growth rate of 7-day-old seedlings (Figure 4) but significantly reduced the biomass and agricultural productivity of wheat in a field experiment (Figure 8). In parallel, Ni reduced the concentration of chlorophyll *a* in shoots (Figure 5) and the expression of *NDOR* in roots (Figure 6), which may indicate a deficiency in the systems of HM detoxification. The first step of xenobiotic detoxification is the biotransformation step, during which active metabolites are formed. An important role in this step is played by cytochrome P450 and related enzymes, such as NADPH-dependent diflavin oxidoreductase (NDOR) [38]. Some PGPRs have been previously shown to neutralize the toxic effects of Ni. *Bacillus subtilis* BM2 inoculation restored wheat biomass and increased chlorophyll content when grown on Ni-contaminated soil [5]. Inoculation with *B. glycinifermentans* IS-2^T^ increased shoot and root elongation compared to uninoculated seeds under both normal and Ni-induced stress [6]. In our experiment, inoculation of *A. picis* B-2897^T^ and *N. irakense* B-2893^T^ seeds increased the shoot weight of 7-day-old seedlings (Figure 4) and yield in the field experiment (Figure 8). *N. irakense* B-2893^T^ increased the expression of *GST* and also prevented the Ni-induced decrease in the expression of the *NDOR* gene (Figure 6), which may indicate the stimulation of detoxification processes in plant roots stimulated by these strains. In addition, *N. irakense* B-2893^T^ reduced the Ni concentration in the dry biomass of 7-day-old wheat seedlings by five times, and in the group of plants whose seeds were inoculated with *A. picis* B-2897^T^, the Ni concentration was below the threshold level (Figure 7), which also indicates the ability of this strain to prevent Ni accumulation in the plant.

Among the studied HMs, Pb had the greatest toxic effect on wheat. It reduced the shoot weight by 8% and the root weight by 18% in 7-day-old seedlings (although the differences with the control were not statistically significant) (Figure 4), as well as the total seed weight in the field experiment by 27%. The root system weight in the field experiment was 66% lower in the soil grown with the addition of Pb (Table S3). It was previously shown that Pb at 585 mg/kg significantly inhibited the germination of wheat seedlings compared to the uninoculated control [29], and at a concentration of 50 μM, it reduced the root growth of *Vicia faba* L. var. *minor* Harz. and caused significant damage to them [8]. We speculate that this negative effect may be due to dysfunction of xenobiotic detoxification systems. We observed a significant decrease in both *GST* and *NDOR* in the roots of 7-day-old wheat seedlings (Figure 6). Wheat seeds inoculated with *A. picis* B-2897^T^ showed improved growth performance 7 days after planting (Figure 4), which was accompanied by an increase in *GST* and *NDOR* expression (Figure 6). Similarly, some PGPRs have been previously shown to improve bacterial tolerance to Pb. Using *Pantotea* sp. Y4-4 to inoculate alfalfa in soil contaminated with Pb resulted in an increase in the total dry weight and root dry weight of the plant [39]. We showed that *A. picis* B-2897^T^ reduced the Pb content in the dry weight of wheat by almost half, and *A. brasilense* B-1547^T^ reduced it by almost four times (Figure 7), which probably provides for the improvement of growth characteristics of wheat seedlings against the background of Pb-induced toxicity. At the same time, in our study, no improvements in agricultural productivity were noted with inoculation with *azospirilla*. The minimum indicators were observed in the group of seeds inoculated with *A. picis* B-2897^T^ and grown in soil with the addition of 1 MAC Pb (Figure 8). In addition, both studied strains did not increase the chlorophyll level but normalized the chlorophyll *a*/chlorophyll *b* ratio closer to 1:1, whereas in non-inoculated wheat grown in soil with the addition of Pb, this ratio was significantly shifted towards chlorophyll *b* (Figure 6). It has been previously reported that photosynthesis rates were reduced due to increased Pb uptake by plants. A sharp decrease in chlorophyll content was observed with increasing amounts of toxic Pb (0, 100, and 400 μM). Higher Pb concentration in plant tissues replaces Mg with Pb, which inhibits enzymatic activity and electron transport in the Calvin cycle and limits chlorophyll synthesis [8]. In general, a shift in the chlorophyll *a*/chlorophyll *b* equilibrium towards chlorophyll *b* is observed under HM-induced stress, particularly Zn. HMs can induce the expression of chlorophyllide *a* oxygenase genes in tobacco, which regulate the conversion of chlorophyll *a* to chlorophyll *b*, which contributes to an increase in the concentration of chlorophyll *b* and more intense work of photosystem II [40]. Probably, in our study, we observed a similar adaptive response of wheat to Pb-induced stress, whereas seed inoculation with *Azospirillum* spp. reduced the stress, which was expressed in a less pronounced adaptive response of the photosynthetic apparatus.

## 5. Conclusions

In general, it is worth noting that three different HMs at a concentration of 1 MAC have opposite effects on wheat growth. Cu stimulates the growth and yield of wheat but slightly inhibits the photosynthetic apparatus, which is neutralized by inoculation of *Azospirillum* spp. Ni and Pb significantly inhibit the growth and yield of wheat, accumulate in plants, reduce the expression of genes responsible for the detoxification of xenobiotics, and change the functionality of the photosynthetic apparatus. Inoculation of seeds with the most resistant *Azospirillum* strains significantly reduces the content of HMs in plants and neutralizes the toxic effect of Ni and, to a slightly lesser extent, Pb, which generally allows them to be considered as potentially effective PGPRs that can be used to increase the productivity of wheat when grown on soils with a high content of HMs. We hypothesize that the use of different *Azospirillum* stains in soils contaminated with different HMs is economically important because it can improve wheat productivity.

## Figures and Tables

**Table 1:** Concentrations of HM salts used in the experiment.

Salt	Concentration Relative to MAC *	Salt Concentration		HM Concentration	
		mg/L	mmol/L	mg/L	mmol/L
Pb(NO_3_)_2_	0.5×	25.5	0.08	15.9	0.08
	1×	51	0.15	31.9	0.15
	2×	102	0.31	63.8	0.31
	5×	255	0.77	159.4	0.77
Cu(SO_4_)·5H_2_O	0.5×	5.85	0.02	1.5	0.02
	1×	11.7	0.05	3	0.05
	2×	23.4	0.09	6	0.09
	5×	58.5	0.23	15	0.23
NiSO_4_·7H_2_O	0.5×	9.5	0.03	2	0.03
	1×	19	0.07	4	0.07
	2×	38	0.14	8	0.14
	5×	95	0.34	20	0.34

* MAC—maximum allowable concentration in soil according to the resolution of the chief state sanitary doctor of the Russian Federation dated 23 January 2006 N 1 On the introduction of hygienic standards GN 2.1.7.2041-06.

**Table 2:** Primers for *Triticum aestivum* L. cDNA used in this work.

Primer Name	Sequence (5′–3′)	Gene ID
*GAPDH* forward	CACCCAACGAAACCCCGTTA	543418
*GAPDH* reverse	AGGAAATCTGGAGCTGCGAC	
*GST* forward	CTTAGACAGGCAGTCAATCCTC	100037529
*GST* reverse	ACCGTAGCCTTGGAGAGGT	
*NDOR* forward	CTTAGACAGGCAGTCAATCCTC	123070686
*NDOR* reverse	GGCGGCTTGATTTTACGAAGT	

**Table 3:** The influence of HMs on the MDA content in bacteria.

Sample	MDA Content, µmol/mg Protein
*A. picis* B-2897^T^	0.04 ± 0.02
*A. picis* B-2897^T^ + Cu	0.33 ± 0.02 *
*A. picis* B-2897^T^ + Ni	0.53 ± 0.02 *
*A. picis* B-2897^T^ + Pb	0.07 ± 0.01
*A. baldaniorum* B-3036^T^	0.22 ± 0.03
*A. baldaniorum* B-3036^T^ + Cu	0.14 ± 0.02
*N. irakense* B-2893^T^	0.16 ± 0.01
*N. irakense* B-2893^T^ + Ni	0.37 ± 0.02 **
*A. brasilense* B-1547^T^	0.03 ± 0.01
*A. brasilense* B-1547^T^ + Pb	0.07 ± 0.01 *

Values are given as mean ± SEM. All measurements were conducted with at least three technical replicates. * *p* < 0.05, ** *p* < 0.01 compared to control (M-U test).

## Data Availability

The data generated and analyzed during the current study are available from the corresponding author upon reasonable request.

## Supplementary Materials

The following supporting information can be downloaded at https://www.mdpi.com/article/10.3390/microorganisms13020334/s1, Table S1: Effect of heavy metals and bacterial inoculation on shoot height of 7-day-old wheat seedlings. Values are given as mean of 10 measurements ± SEM. * *p* < 0.05 relative to control and ** *p* < 0.01 relative to control; Table S2: Effect of heavy metals and bacterial inoculation on MDA concentration (μM/g.d.m.) in shoots and roots of wheat seedlings. Values are given as mean of 10 measurements ± SEM; Table S3: The effect of heavy metals and bacterial inoculation on wheat yield.

## Abbreviations

The following abbreviations are used in this manuscript:

HM	heavy metal
ROS	reactive oxygen species
PGPRs	plant growth-promoting rhizobacteria
MAC	maximum allowable concentration
MDA	malondialdehyde
NDOR	NADPH-dependent diflavin oxidoreductase
GST	glutathione-S transferase
