# Supplementary material for: Effect of Bacteria from the Genus Azospirillum on Oxidative Stress Levels in Wheat Triticum aestivum L. in the Presence of Copper, Nickel, and Lead
Source: Microorganisms. 2025 Feb 4;13(2):334. doi: 10.3390/microorganisms13020334 (PMC11858639; doi:10.3390/microorganisms13020334)
Supplement: Supplementary file 1 [file microorganisms-13-00334-s001.zip › microorganisms-3456288-supplementary.pdf]

Effect of bacteria from the genus *Azospirillum* on oxidative stress levels in wheat *Triticum aestivum* L. in the presence of copper, nickel and lead

Maria V. Gureeva, Marina S. Kirillova, Veronika A. Trandina, Vera A. Kryukova, Anna A. Eremina, Alina A. Alimova, Margarita Y. Grabovich and Artem P. Gureev

Supplementary Materials

Table S1. Effect of heavy metals and bacterial inoculation on shoot height of 7-day-old wheat seedlings. Values are given as mean of 10 measurements ± SEM. * - $p<0.05$ relative to control, ** - $p<0.01$ relative to control.

Experimental group	Height of shoots, cm
Control	15.31±0.53
Pb	14.67±1.19
Pb + *A. picis* B-2897^T	16.53±0.51
Pb + *A. brasilense* B-1547^T	16.31±0.65
Cu	14.59±0.73
Cu + *A. picis* B-2897^T	17.7±0.55
Cu + *A. baldaniorum* B-3036^T	16.5±0.44
Ni	15.08±1.45
Ni + *A. picis* B-2897^T	18.27±0.5**
Ni + *N. irakense* B-2893^T	15.33±0.003
A. picis B-2897^T	16.19±0.46
A. baldaniorum B-3036^T	17.25±0.86
N. irakense B-2893^T	17.96±0.66*
A. brasilense B-1547^T	17.71±0.59*

Table S2. Effect of heavy metals and bacterial inoculation on MDA concentration (µM/g.d.m.) in shoots and roots of wheat seedlings. Values are given as mean of 10 measurements ± SEM.

Experimental group	MDA content in shoots	MDA content in roots
Control	6.3 ± 0.8	5.1 ± 0.6
A. picis B-2897^T	3.6 ± 0.5	3.8 ± 0.5
A. baldaniorum B-3036^T	8.1 ± 0.1	5.9 ± 1.6
N. irakense B-2893^T	3.4 ± 0.1	5.4 ± 1.1
A. brasilense B-1547^T	4.8 ± 0.4	8.1 ± 1.6
Cu + *A. picis* B-2897^T	3.3 ± 0.6	4.8 ± 0.0
Cu + *A. baldaniorum* B-3036^T	4.8 ± 0.1	4.3 ± 0.1
Ni + *A. picis* B-2897^T	4.4 ± 0.1	5.9 ± 0.5
Ni + *N. irakense* B-2893^T	3.1 ± 0.1	2.7 ± 1.6
Pb + *A. picis* B-2897^T	2.0 ± 0.2	8.6 ± 3.2
A. brasilense B-1547^T	4.5 ± 1.2	3.8 ± 0.5

Table S3. The effect of heavy metals and bacterial inoculation on wheat yield

Group	Weight of all seeds, g	Weight of 1000 seeds, g	Root weight, g	Straw weight, g	Number of ears per plot
Control	141.55	35.15	111.5	350	345
Cu	163.3	40.3	226	494	281
Cu + *A. picis* B-2897^{T}	139.8	34.5	203	408	293
Cu + *A. baldaniorum* B-3036^{T}	191.8	39.6	268	524	354
Ni	49.8	30.6	38	414	387
Ni + *A. picis* B-2897^{T}	157.6	40.8	98	460	357
Ni + *N. irakense* B-2893^{T}	116.4	37.1	390	465	374
Pb	103.4	37.3	40	430	358
Pb + *A. picis* B-2897^{T}	47.7	26.6	69	375	394
Pb + *A. brasilense* B-1547^{T}	125.1	34.2	37	290	332
A. picis B-2897^{T}	162.4	36.6	141	431	375
A. baldaniorum B-3036^{T}	222	36	118	408	431
N. irakense B-2893^{T}	166.6	35.9	91	415	406
A. brasilense B-1547^{T}	137	36.6	58	114	423
